# Supplementary material for: [99mTc]Tc-PSMA-T4—Novel SPECT Tracer for Metastatic PCa: From Bench to Clinic
Source: Molecules. 2022 Oct 25;27(21):7216. doi: 10.3390/molecules27217216 (PMC9658561; doi:10.3390/molecules27217216)
Supplement: Supplementary file 1 [file molecules-27-07216-s001.zip › molecules-1946796-supplementary.pdf]

Supplementary materials
to the manuscript "*[^{99m}Tc]Tc-PSMA-T4 – novel SPECT tracer for metastatic PCa: from bench to clinic*"

A: Spectral NMR confirmation of the structure of the sample PSMA-T4 supplied by NCNR

Multinuclear MR spectra were made to confirm the structure of the compound (Scheme S1):

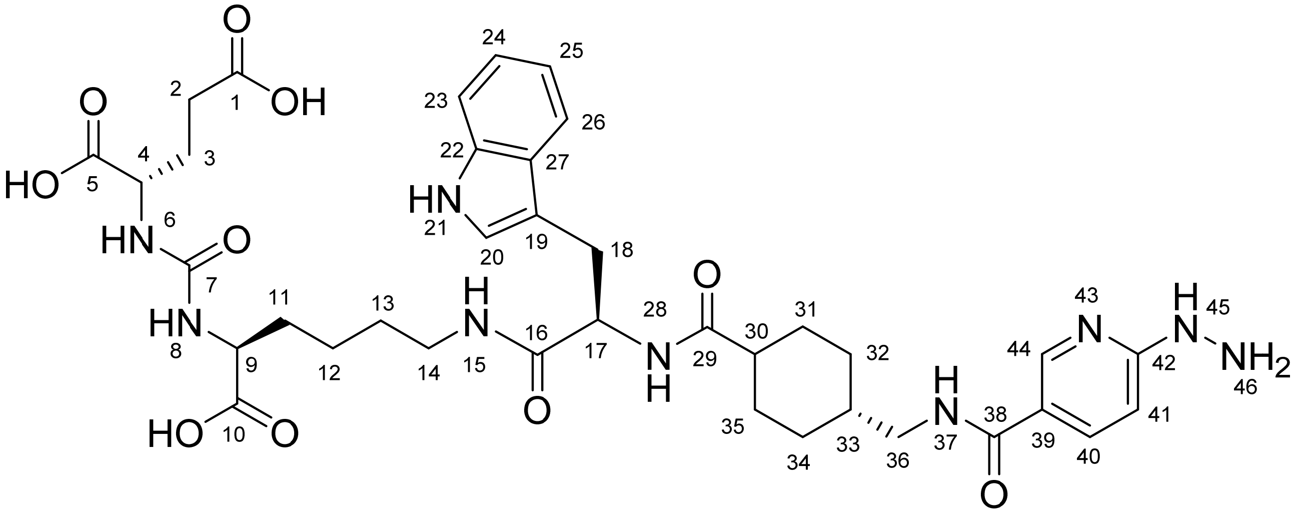

Scheme S1. Numbering of PSMA-T4 for the 1H/13C/15N NMR signals assignment.

Position number	δ (^{15}N) [ppm]	δ (^{13}C) [ppm]	δ (^{1}H) [ppm]	multiplicity [(c)]	*J*(H,H) couplings [Hz]
**1**	-----	173.70	-----	-----	-----
**OH at C1**	-----	-----	~12.0[a]	s (br) [b]	
**2**	-----	29.88	2.24	m	-----
**3**	-----	27.51	1.71/1.92	m(ov)/m	-----
**4**	-----	51.64	4.10	m	-----
**5**	-----	174.16	-----	-----	-----
**OH at C5**	-----	-----	~12.0[a]	s (br) [b]	
**6**	-294.0	-----	6.33	d	8.4
**7**	-----	157.29	-----	-----	-----
**8**	-292.9	-----	6.29	d	8.1
**9**	-----	52.26	4.03	m	-----
**10**	-----	174.54	-----	-----	-----
**OH at C10**	-----	-----	~12.0[a]	s (br) [b]	
**11**	-----	31.70	1.48/1.62	m/m	-----
**12**	-----	22.54	1.23	m(ov)	-----
**13**	-----	28.66	1.32	m(ov)	-----
**14**	-----	38.38	2.99	m(ov)	-----
**15**	-264.8	-----	7.86	t	5.3
**16**	-----	171.43	-----	-----	-----
**17**	-----	53.20	4.44	m	-----
**18**	-----	28.05	2.90/3.04	dd/m(ov)	8.7; 14.5/-----
**19**	-----	110.24	-----	-----	-----

**20**	----	123.46	7.08	d	1.5
**21**	-249.5	-----	10.75	s	-----
**22**	-----	135.97	-----	-----	-----
**23**	-----	111.18	7.30	d	7.9
**24**	-----	120.76	7.03	t	7.5; 7.3
**25**	-----	118.07	6.95	t	7.3; 7.6
**26**	-----	118.51	7.57	d	8.0
**27**	-----	127.37	-----	-----	-----
**28**	-260.0	-----	7.78	d	8.2
**29**	-----	174.88	-----	-----	-----
**30**	-----	43.69	2.10	m	-----
**31**	-----	28.66	1.16/1.56	m(ov)/m(ov)	-----
**32**	-----	29.79	0.87/1.70	m(ov)/m(ov)	-----
**33**	-----	37.03	1.44	m(ov)	-----
**34**	-----	29.70	0.90/1.74	m(ov)/m(ov)	----
**35**	-----	28.48	1.24/1.70	m(ov)/m(ov)	-----
**36**	-----	45.31	3.08	m(ov)	-----
**37**	-270.3	-----	8.46	t	5.4
**38**	-----	163.74	-----	-----	-----
**39**	-----	121.90	-----	-----	-----
**40**	-----	138.09	8.12	dd	1.9; 8.9
**41**	-----	108.93	6.88	d	9.0
**42**	-----	156.70	-----	-----	-----
**43**	[d]	-----	-----	-----	-----
**44**	-----	143.72	8.58	s	-----
**45**	[d]	-----	9.75	s (br)	-----
**46**	[d]	-----	~3.5[e]	s (br)	

a – broad average signal for all protons of OH groups, b – broad averaged singlet, c – multiplicity: [s – singlet, d – doublet, dd- doublet of doublets, t – triplet, m – multiplet, m(ov) – overlapping multiplets s(br) – very broad signal], d – not recorded in the ^{1}H-^{15}N HMBC experiments, probably due the exchange process, e – averaged signal of exchangeable protons of NH_2 group and H_2O

1. **Equipment and reagents**
   1.1. **Instruments:**
   The NMR spectra were recorded at Varian VNMRS-600 spectrometers equipped with a 5-mm PFG AutoXID($^{1}H/X^{15}N$-^{31}P) probe.
   2.1 Reagents and sample preparation

The sample was dissolved in 0.6 ml of DMSO-D6 – 99.80 %D Euriso-top lot: Q22681, batch:0817H.

2. **Methodology**

The structure of the compound (Scheme S1) was determined by interpretation of the one-dimension ^{1}H, ^{13}C, DEPT-135 spectra, two-dimension homonuclear COSY, TOCSY, ROESY and heteronuclear ^{1}H-^{13}C HSQC, HSQC-TOCSY, HMBC NMR spectra. Proton connectivities were derived from COSY, TOCSY and ROESY spectra. The ^{13}C resonances corresponding to carbons with directly attached protons were assigned using HSQC and HSQC-TOCSY spectra. HMBC spectra were used to assign resonances of the quaternary carbons and to validate the connectivities established by the other spectra. Results of both ^{1}H-^{15}N correlations (HSQC and HMBC) were used for confirmation of character of nitrogen atoms in the compound studied.

The spectra were measured at 298K in deuterated dimethyl sulfoxide (DMSO) and were calibrated using appropriate signals of DMSO [δ = 2.50 ppm (^{1}H NMR), and δ = 39.5 ppm (^{13}C NMR)] as standard for $^{1}H/^{13}C$ NMR spectra, respectively. The ^{15}N spectra were calibrated using nitromethane as external

standard for which δ = 0.0 ppm, and the ^{15}N chemical shifts were determined on a basis of 2D ^{1}H-^{15}N HSQC/HMBC spectra. Concentration of the solution used for measurements was *ca.* 20 mg of PSMA-T4 in 0.6 ml of solvent. In Figures 1 – X all basic spectra for recoded experiments are presented.

The experiments were performed in the following conditions:

^{1}H spectra - 64 transients, relaxation delay: 1.0 s, pulse width: 2.3 μs (30^0), 48 K data points zero-filled to 128 K, spectral width *ca.* 7600 Hz

{^{1}H}^{13}C NMR - 24000 transients, relaxation delay: 0.5 s, pulse width:4.2 μs (30^0), 90 K data points zero-filled to 128 K, spectra width *ca.* 38000 Hz.

DEPT-135, 1024 transients, relaxation delay: 1.0 s, pulse width (^{1}H): 6.9/13.8 μs ($90^0/180^0$), pulse width (^{13}C): 12.6 μs (90^0), 64 K data points zero-filled to 128K, spectral width *ca.* 38000 Hz

COSY – spectral widths 7600 Hz in both dimensions, 512 complex point in in t_2 and t_1, 2 scans per increment, relaxation delay 1s,

TOCSY – spectral widths 7600 Hz in both dimensions, 512 complex points in t_2 and t_1, 2 scans per increment, relaxation delay 1s and spin-lock times: 18 ms and 80 ms, respectively,

ROESY– spectral widths 7600 Hz in both dimensions, 1024 complex points in t_2, 512 complex points in t_1, 8 scans per increment, relaxation delay 1s and mixing time 300 ms,

^{1}H-^{13}C/(^{1}H-^{15}N) HSQC - spectral widths 7600 Hz in F2 and 25600 Hz in F1 (^{13}C) or 8000 Hz (^{15}N), 1024 complex points in t_2, 2048 complex points in t_1, 2 or 8 (^{15}N) scans per increment, relaxation delay 1s,

^{1}H-^{13}C HSQC-TOCSY - spectral widths 7600 Hz in F2 and 25600 Hz in F1, 1024 complex points in t_2, 2048 complex points in t_1, 2 scans per increment, relaxation delay 1s and spin-lock time 80 ms,

^{1}H-^{13}C/(^{1}H-^{15}N) HMBC - spectral widths 7600 Hz in F2 and 27600 Hz in F1 (^{13}C) or 22000 Hz (^{15}N), 1024 complex points in t_2, 2048 complex points in t_1, 16 or 32 (^{15}N) scans per increment.

The data were processed with linear prediction in t_1 followed by zero-filling in both dimensions. Gaussian weighting functions were applied in both domains prior to Fourier transformation. In the cases where signal to noise was sufficient, the use of sine weighting functions facilitated a better spectra resolution.

3. Results

Careful analysis of different NMR experiments described above confirm the structure of the compound studied presented in Scheme S1. However nitrogen atoms N43, N45 and N46 can exist in other forms due to protons exchange.

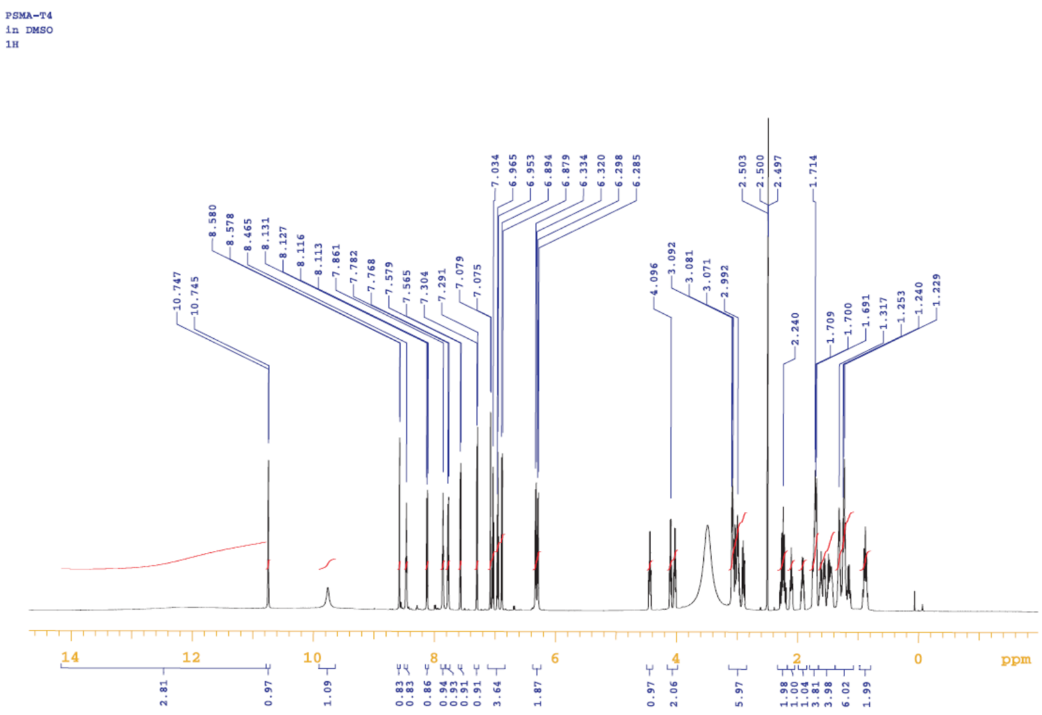

Figure S1: The ¹H NMR spectrum of PSMA-T4 in DMSO.

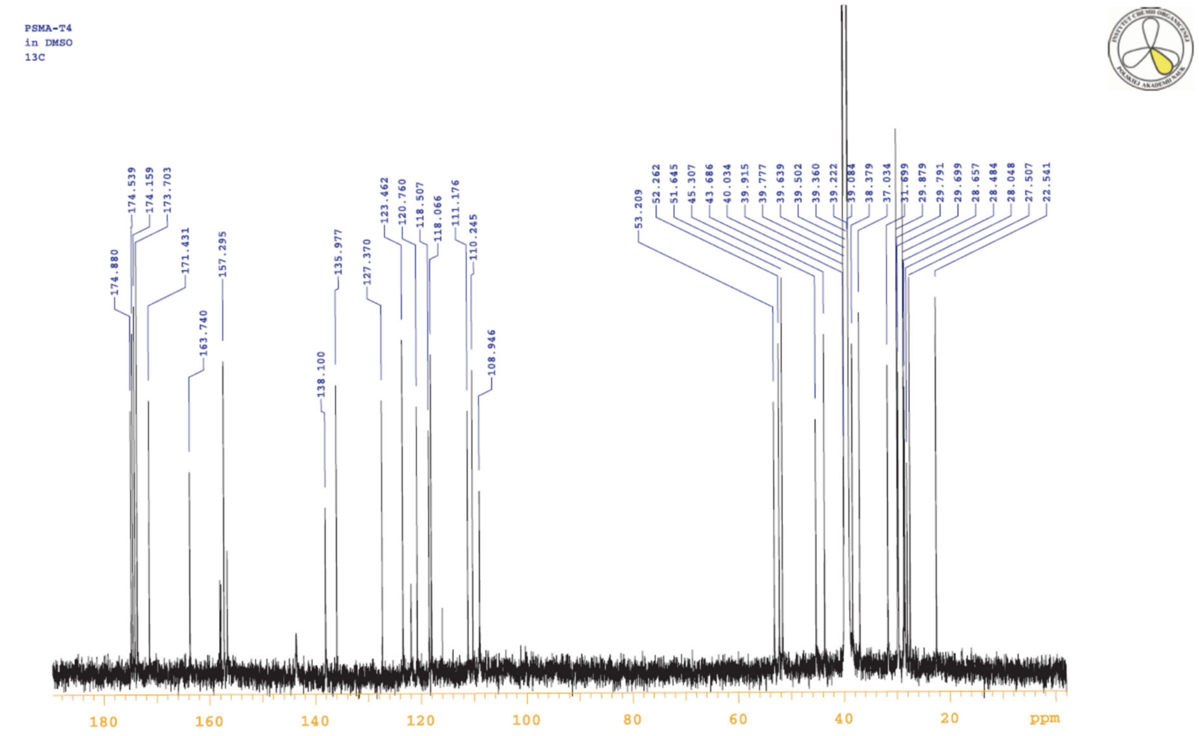

Figure S2: The ^{13}C NMR spectrum of PSMA-T4 in DMSO.

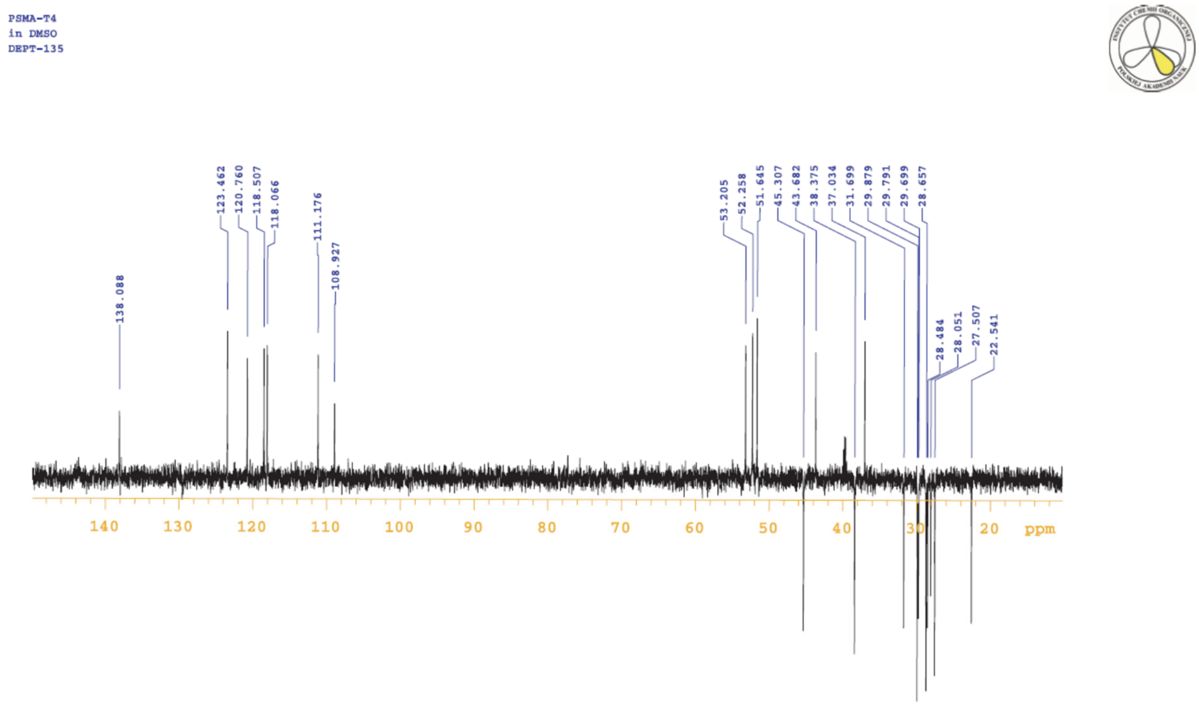

Figure S3: The Dept-135 spectrum of PSMA-T4 in DMSO.

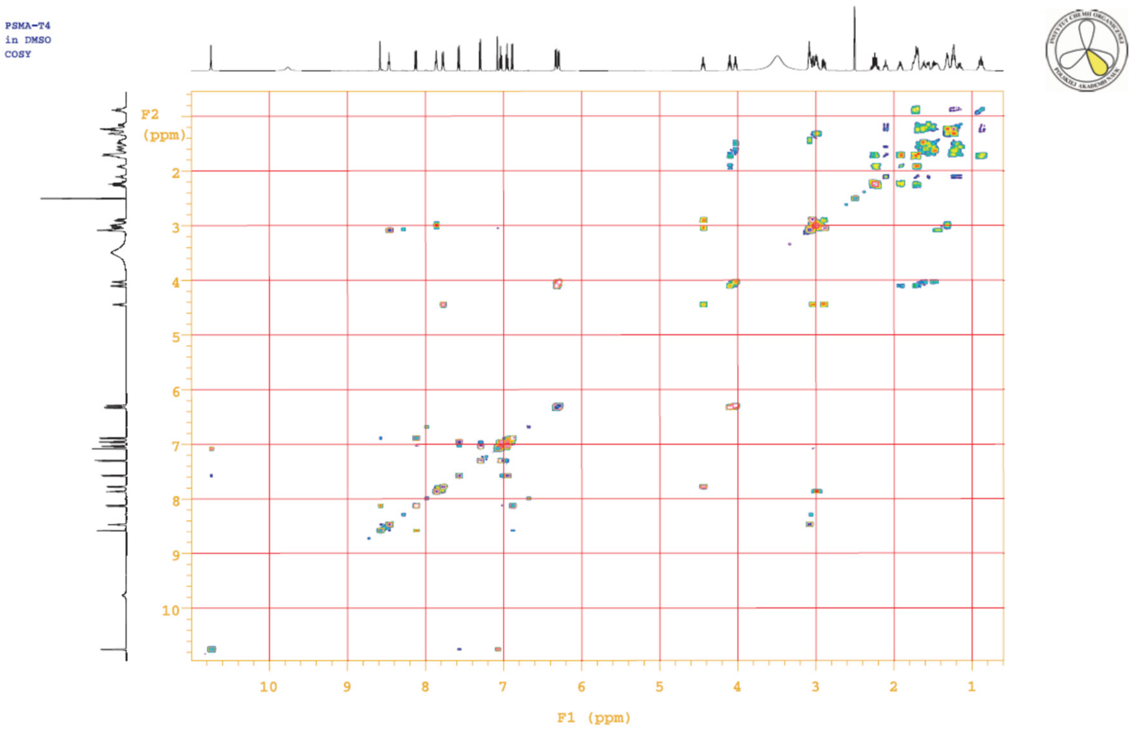

Figure S4: COSY spectrum of PSMA-T4 in DMSO.

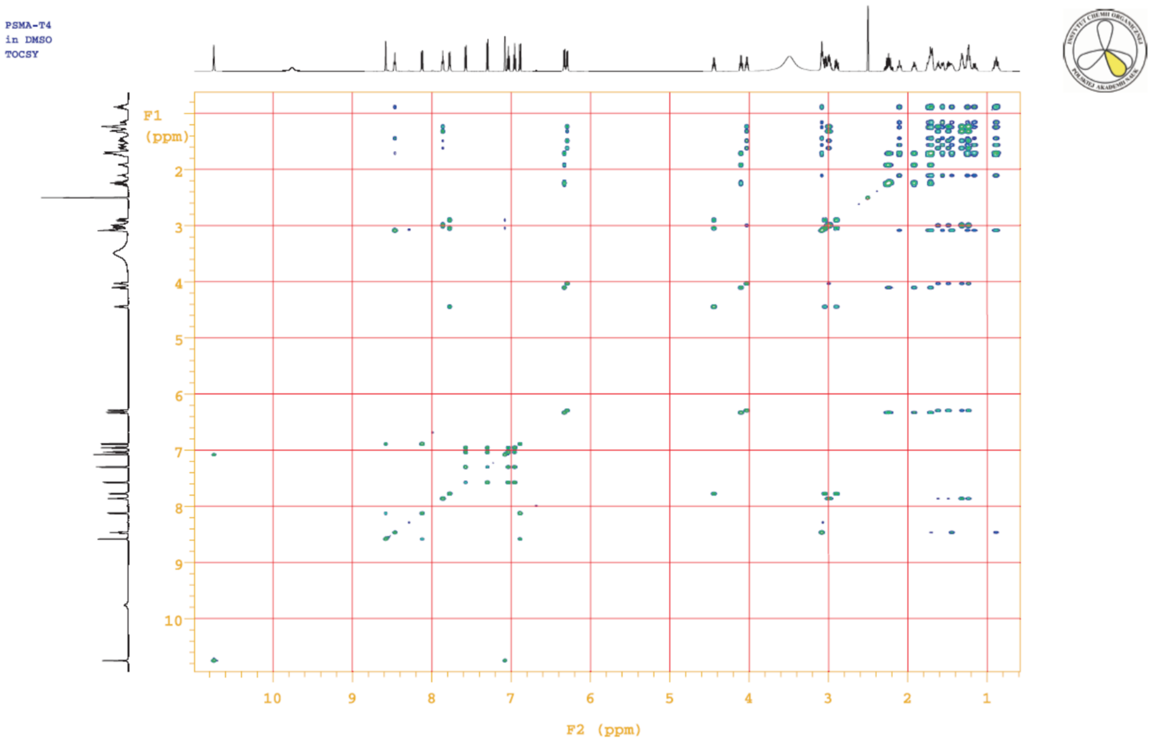

Figure S5: TOCSY spectrum of PSMA-T4 in DMSO.

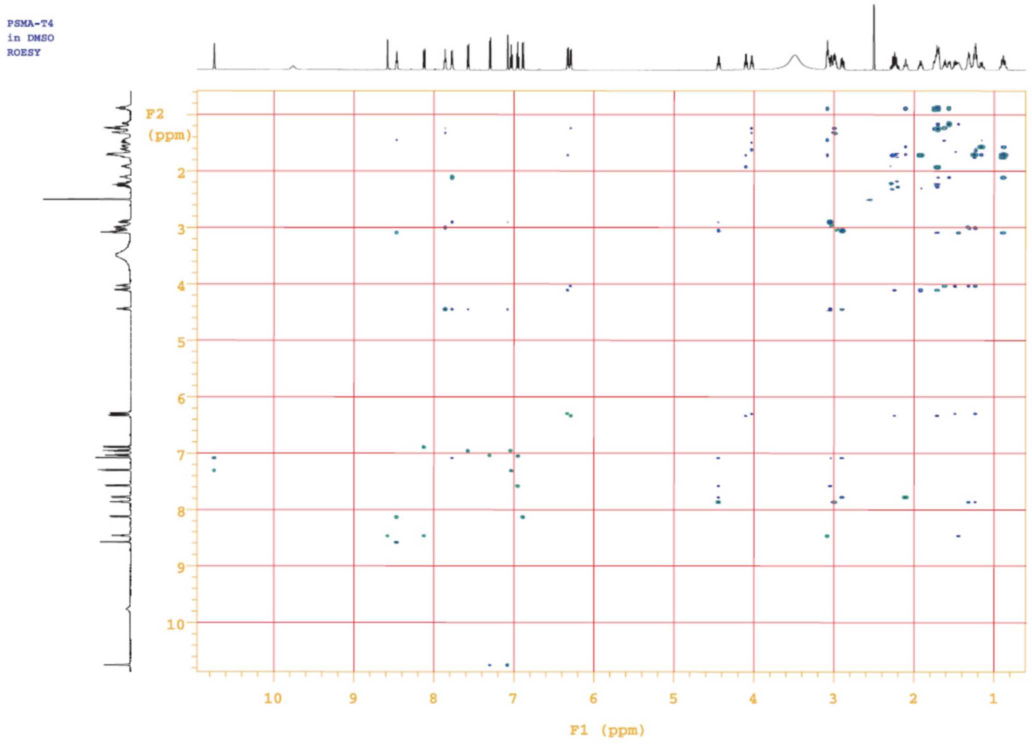

Figure S6:. ROESY spectrum of PSMA-T4 in DMSO.

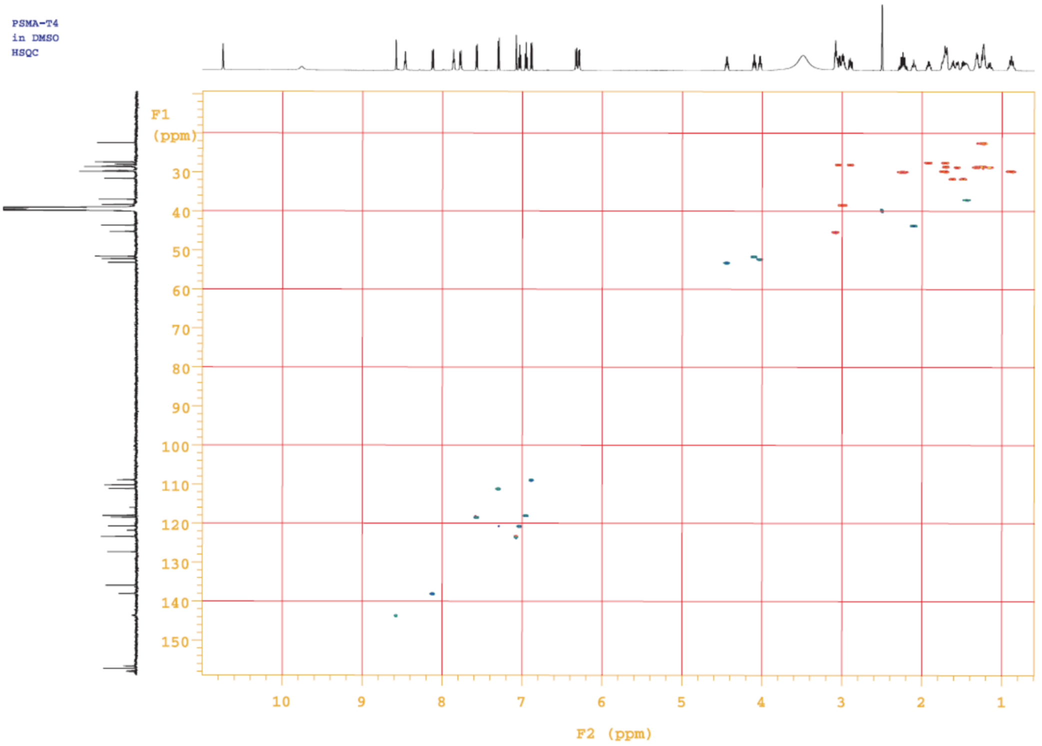

Figure S7:. The ^{1}H-^{13}C HSQC spectrum of PSMA-T4 in DMSO.

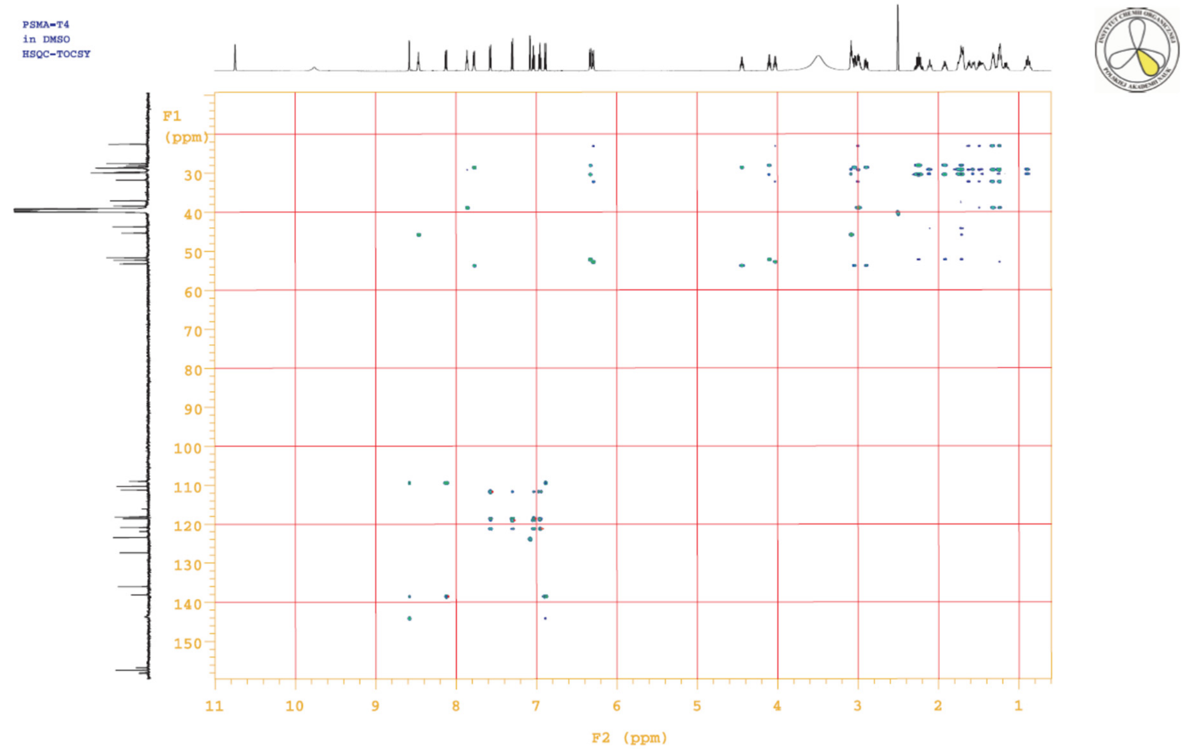

Figure S8: The ^{1}H-^{13}C HSQC-TOCSY spectrum of PSMA-T4 in DMSO.

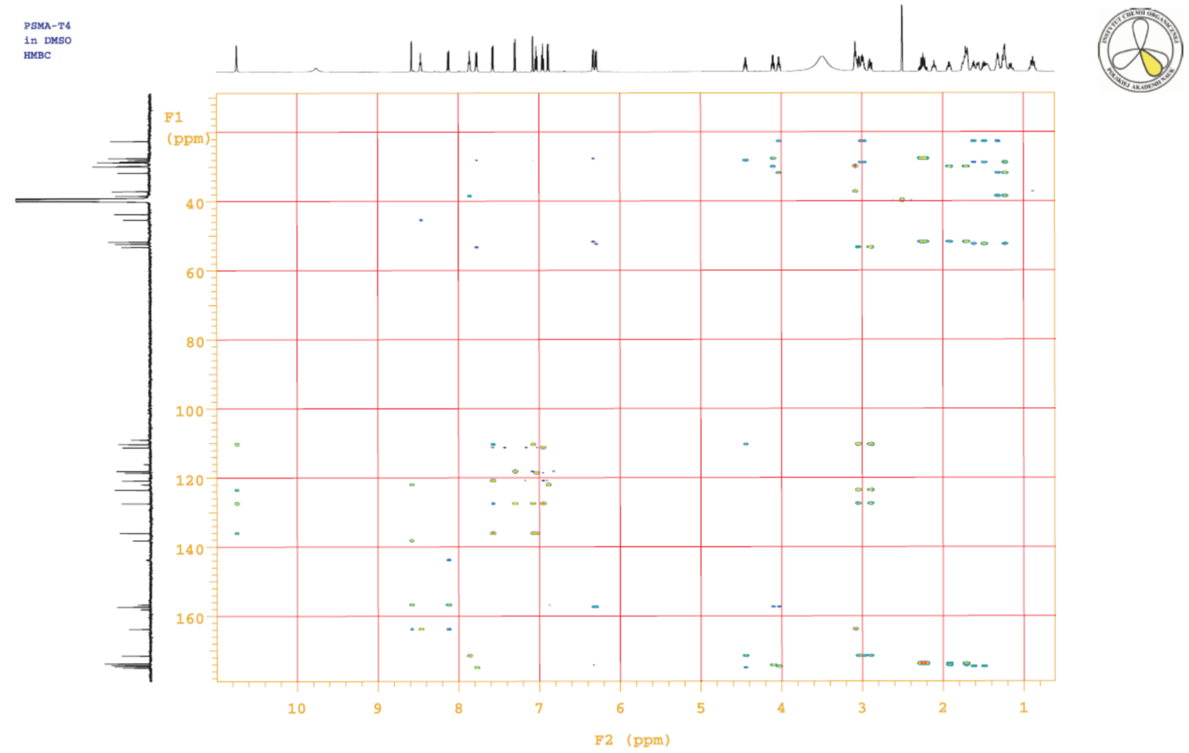

Figure S9: The ^{1}H-^{13}C HMBC spectrum of PSMA-T4 in DMSO.

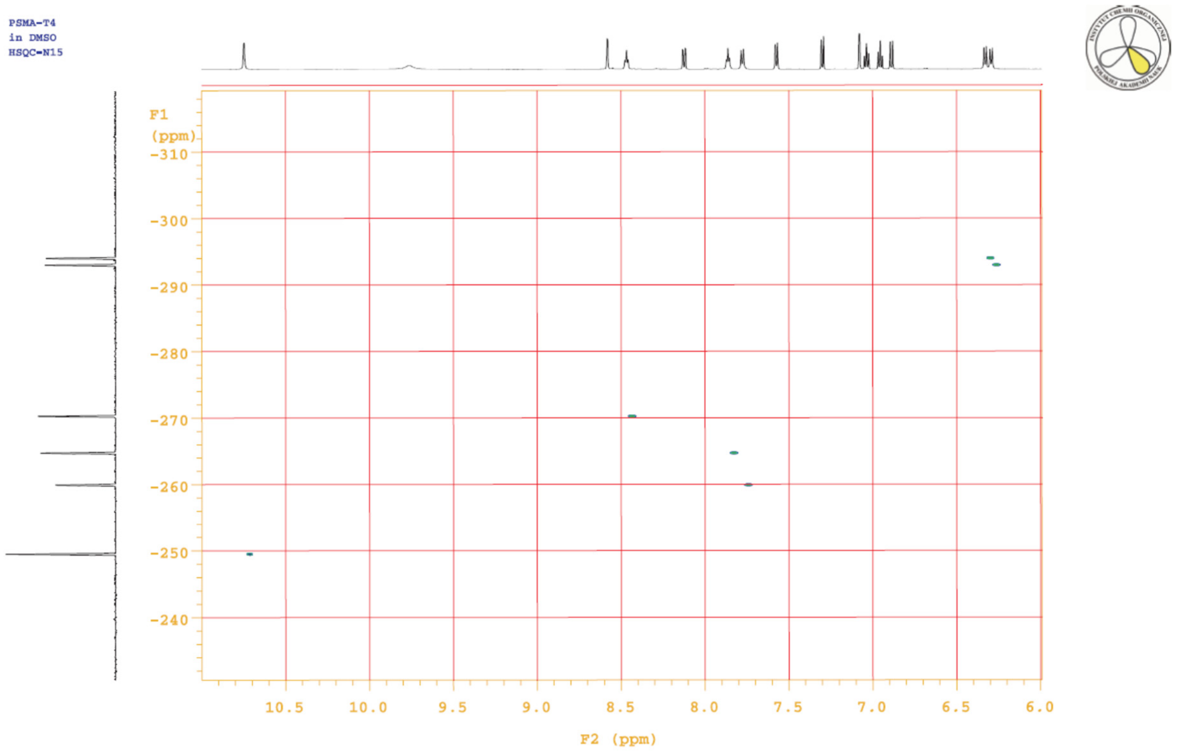

Figure S10:. The ^{1}H-^{15}N HSQC spectrum of PSMA-T4 in DMSO.

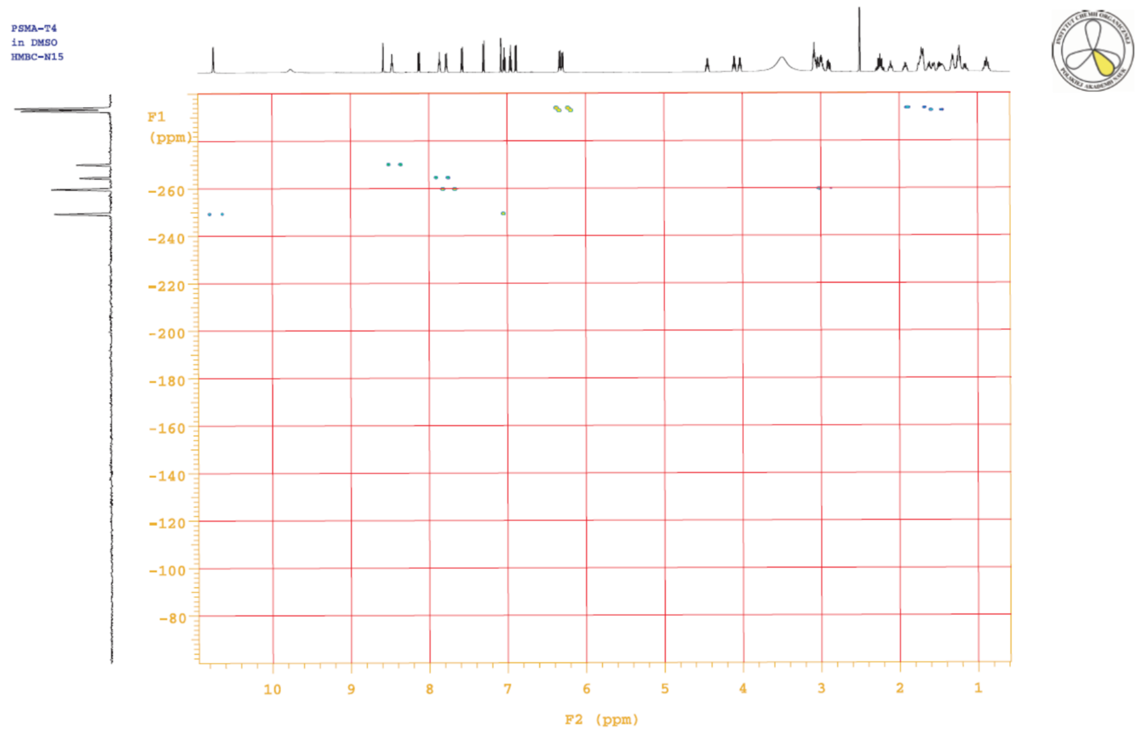

Figure S11:. The ^{1}H-^{15}N HMBC spectrum of PSMA-T4 in DMSO.

B. Kit Preformulation studies

Table S1. Influence of tricine and EDDA quantity (added separately) on radiochemical purity [^{99m}Tc]Tc-PSMA-T4 obtained with 0.35 GBq ^{99m}Tc eluate labeling in a volume of 2 mL at constant parameters of 23 μg PSMA-T4, 50 μg $SnCl_2$, $2H_2O$ and incubation 15 min at 95°C.

EDDA [mg]	Tricine [mg]	RCP (%)
1	-	60.7
3	-	56.5
5	-	57.2
7	-	62.8
10	-	69.0
-	10	31.4
-	25	55.2
-	50	54.9
-	70	35.2
-	100	57.8

Table S1. The effect of quantity of co-ligands on radiochemical purity of [^{99m}Tc]Tc-PSMA-T4.

EDDA [mg]	Tricine [mg]	RCP (%)
1	50	41.5
3	50	44.4
5	50	97.2
7	50	97.5
10	50	96.8
5	10	95.0
5	25	88.5
5	50	97.5
5	70	97.5
5	100	97.4

Table S3. Radiochemical purity of [^{99m}Tc]Tc- PSMA-T4 with varying amounts of stannous chloride.

$SnCl_2$, $2H_2O$ [μg]	RCP [%]
10	97.7
30	97.9
50	97.6
100	95.8
150	95.2

Table S2. Relationship between the activity of $^{99m}TcO_4^-$ added to 23 μg of PSMA-T4, 50 mg Tricine, 5 mg EDDA and 50 μg $SnCl_2$, $2H_2O$.

Activity of $^{99m}TcO_4^-$ [GBq]	RCP [%] 0h	RCP (%) 3h
0.3	96.7	96.0
0.74	96.8	96.2
1.0	97.8	95.2
1.5	95.6	91.6
2.2	89.9	n.d.

C. In vivo studies

Table S5: The calculated ***p*-valu**e based on ordinary two-way ANOVA and Tukey's multiple comparisons test, with a single pooled variance.

	iPSMA vs			
**Organ**	**PSMA-T1**	**PSMA-T2**	**PSMA-T3**	**PSMA-T4**
blood	>0,9999	>0,9999	>0,9999	>0,9999
thyroid	0,9898	0,9898	0,9898	0,9898
heart	>0,9999	>0,9999	>0,9999	>0,9999
lung	>0,9999	>0,9999	>0,9999	>0,9999
liver	>0,9999	>0,9999	>0,9999	>0,9999
spleen	0,8012	0,8012	0,8012	0,8012
pancreas	0,9989	0,9989	0,9989	0,9989
**kidneys**	**<0,0001**	**<0,0001**	**<0,0001**	**<0,0001**
intestine	0,9999	0,9999	0,9999	0,9999
stomach wall	>0,9999	>0,9999	>0,9999	>0,9999
femur	>0,9999	>0,9999	>0,9999	>0,9999
muscle	>0,9999	>0,9999	>0,9999	>0,9999
**urine**	**<0,0001**	**<0,0001**	**<0,0001**	**<0,0001**

Table S6: The calculated p-value in renal uptake between ^{99m}Tc-labeled PSMA ligands.

	**p-value**
PSMA-T1 vs. PSMA-T2	<0,0001
PSMA-T1 vs. PSMA-T3	<0,0001
PSMA-T1 vs. PSMA-T4	<0,0001
PSMA-T2 vs. PSMA-T3	<0,0001
PSMA-T2 vs. PSMA-T4	<0,0001
PSMA-T3 vs. PSMA-T4	0,1322

Table S7: Comparison of biodistribution study of [^{99m}Tc]Tc-PSMA-T4 complex in BALB/c Nude mice (n=5) 4h after intravenous administration of different doses (%ID/g. mean±SD).

	**[^{99m}Tc]Tc-PSMA-T4 0.2 μg n=5**	**[^{99m}Tc]Tc-PSMA-T4 1.2 μg n=3**	**[^{99m}Tc]Tc-PSMA-T4 0.2 μg + 22 μg PSMA-T4 n=5**
blood	0.16±0.03	0.10±0.03	0.07±0.02
thyroid	0.35±0.10	0.06±0.06	0.18±0.11
liver	0.11±0.02	0.08±0.02	0.08±0.02
spleen	1.59±0.27	0.77±0.24	0.08±0.06
kidney	36.23±3.52	14.46±1.35	1.17±0.58
intestine	1.41±1.17	0.89±0.15	1.72±0.74
stomach wall	0.21±0.09	0.02±0.01	0.11±0.05
muscle	0.11±0.05	0.04±0.02	0.01±0.02
tumour	17.46±3.21	11.57±2.41	0.83±0.53
urine [%ID]	91.44±4.44	60.93±20.24	96.60±0.90
